# Spotlight on Isl1: A Key Player in Cardiovascular Development and Diseases

**DOI:** 10.3389/fcell.2021.793605

**Published:** 2021-11-25

**Authors:** Jie Ren, Danxiu Miao, Yanshu Li, Rui Gao

**Affiliations:** ^1^ Xiamen Cardiovascular Hospital, Xiamen University, Xiamen, China; ^2^ Department of Toxicology, College of Public Health, Harbin Medical University, Harbin, China

**Keywords:** Isl1, cardiac transcription factor, cardiovascular development, second heart field progenitors, embryonic development

## Abstract

Cardiac transcription factors orchestrate a regulatory network controlling cardiovascular development. Isl1, a LIM-homeodomain transcription factor, acts as a key player in multiple organs during embryonic development. Its crucial roles in cardiovascular development have been elucidated by extensive studies, especially as a marker gene for the second heart field progenitors. Here, we summarize the roles of Isl1 in cardiovascular development and function, and outline its cellular and molecular modes of action, thus providing insights for the molecular basis of cardiovascular diseases.

## Introduction

The heart is the first organ to form during mammalian embryogenesis. The formation of the heart begins at the gastrulation stage from mesoderm progenitor cells migrated from the primitive streak (Buckingham et al., 2005). These mesoderm progenitor cells segregate into two populations during heart development, referred to as the first heart field (FHF) and the second heart field (SHF) (Buckingham et al., 2005). Cardiac progenitors in the FHF firstly differentiate to form the primitive heart tube, while the SHF is constituted by a highly proliferative progenitor population, which adds gradually to the arterial and venous poles, contributing to the expansion of the early heart tube (Cai et al., 2003; Evans et al., 2010; Vincent and Buckingham, 2010). The FHF is the source of the early left ventricle (LV) whereas the SHF gives rise to the outflow tract (OFT) and the right ventricle (RV), and both populations contribute to the atria (Evans et al., 2010; Vincent and Buckingham, 2010). During heart development, multiple transcription factors orchestrate a spatiotemporal regulatory network to ensure the fine regulation of cardiogenesis (Bruneau, 2002). Islet1 (Isl1) is one of the most important transcription factors, playing key roles in cardiovascular development.

The story of Isl1 began in 1990, when Karlsson et al. isolated a cDNA which encodes a protein with the ability of binding Rat insulin I gene enhancer (Karlsson et al., 1990). In human, ISL1 localizes in chromosome 5, containing 6 exons (Tanizawa et al., 1994; Riggs et al., 1995). ISL1 is a LIM-homeodomain transcription factor, consisting of two LIM domains at the N-terminus, one homeodomain in the central region, and a C-teminus region as well. LIM domains are responsible for protein-protein interactions, while the homeodomain functions as a DNA binding domain (Scnchez-Garcia et al., 1993; Rétaux and Bachy, 2002). Interestingly, ISL1 prevents itself from DNA binding by an intramolecular interaction which can be released when ISL1 binds to its protein partners, such as LDB1 (Scnchez-Garcia et al., 1993; Gadd et al., 2013).

Isl1 plays critical roles in multiple biological processes, such as islet cell differentiation, motor neuron generation, as well as cardiovascular development (Pfaff et al., 1996; Ahlgren et al., 1997; Cai et al., 2003; Liang et al., 2011; Gao et al., 2019). In this review, we will summarize not only the roles of Isl1 in cardiovascular development and diseases, but also significant advances in the cellular and molecular mechanisms of action of Isl1 during cardiogenesis, therefore providing insights for the molecular basis of Isl1 in cardiovascular diseases.

### The Expression of Isl1

In the past decades, a variety of studies have demonstrated that Isl1 plays essential roles during embryogenesis. Visualization of Isl1 expression profile provides insight into its potential roles during development. Previous studies have revealed expression of Isl1 in multiple tissues and cell types, including islet cells (Karlsson et al., 1990; Ahlgren et al., 1997), neurons (Thor et al., 1991; Pfaff et al., 1996; Liang et al., 2011), cardiac progenitor cells (CPCs) (Cai et al., 2003), incisor epithelium (Mitsiadis et al., 2003) and hindlimb bud (Yang et al., 2006; Tahara et al., 2018), and so on. Here, we will mainly focus on the expression of Isl1 in cardiovascular system.

During mouse heart development, Isl1 expression can be detected as early as E7.0, at the cardiac crescent stage, locating contiguous with, but medial and dorsal to, MLC2a-expressing cells (Cai et al., 2003; Sun et al., 2007a). At E8.0, Isl1 is expressed at the pharyngeal mesoderm dorsal to the heart tube (the SHF progenitors) (Cai et al., 2003; Zhuang et al., 2013). Between E8.5 and E9.0, when SHF progenitors migrate and join the arterial and venous poles, Isl1 was observed to be actively expressed in most cells of the RV and OFT, and partially expressed in the atria, but not in the rest of the myocardium (Cai et al., 2003; Sun et al., 2007b). Then the expression of Isl1 in the heart decreases while CPCs undergo differentiation and becomes progressively confined to a subdomain within the right atria, in the region of the cardiac pacemaker (Laugwitz et al., 2005; Sun et al., 2007b). At the same time, Isl1 starts to express in many other tissues, such as in the subregions of midbrain and basal forebrain, spinal motor neurons, cranial ganglia, dorsal root ganglia, posterior hindlimb and pancreatic epithelium (Ahlgren et al., 1997; Sun et al., 2007b; Zhuang et al., 2013). By E14.5, Isl1 expression only persists in a subset of cells in the heart, including subdomains within the OFT, RV, venous valves, aorta, pulmonary artery, atrial septum, and in regions of sinoatrial node (SAN), atrioventricular node (AVN), and in clusters of cells of cardiac ganglia (Sun et al., 2007b). From postnatal stage to adulthood, Isl1 expression is still observed in several cell types of the heart, which are localized in the SAN, very few if any in the AVN, at the base of the aorta and pulmonary artery and in cardiac ganglia (Moretti et al., 2006; Sun et al., 2007b; Weinberger et al., 2012; Zhou et al., 2019).

Importantly, the expression pattern of ISL1 in human fetal heart matches the known contribution of Isl1^+^ precursors to SHF derivatives in the mouse heart (Laugwitz et al., 2005; Bu et al., 2009; Genead et al., 2010b; Yang et al., 2013). In addition, a number of studies have produced clear evidence that, in accordance with its expression profile, Isl1 marks the SHF progenitors during cardiogenesis (Cai et al., 2003; Moretti et al., 2006; Bu et al., 2009; Gao et al., 2019), even though Isl1 was found to be transiently detected in a small number of cells in the FHF (Ma et al., 2008). Interestingly, there have been reports of small numbers of Isl1^+^ cells being detected in the hearts of adult mice (Genead et al., 2010a; Khattar et al., 2011; Weinberger et al., 2012; Zhou et al., 2019), but there is growing evidence to suggest that these cells may not be functioning as CPCs (Weinberger et al., 2012; Zhou et al., 2019).

### The Functions of Isl1

In 2003, Evans group reported that Isl1 knockout (KO) mice are embryonic lethal (die at approximately E10.5), completely missing the OFT, RV and much of the atria (Cai et al., 2003). This study clearly showed that Isl1 is essential for embryonic heart development. Moreover, Isl1 was further demonstrated to be required for the proliferation, survival and migration of Isl1^+^ CPCs (Cai et al., 2003). Shortly thereafter, a series of publications from Chien group took these findings a significant step forward, identifying that Isl1^+^ CPCs are multipotent, which are capable of differentiating into all the three major cell types in the heart, including cardiomyocytes, endothelial cells and smooth muscle cells (Moretti et al., 2006; Bu et al., 2009).

Following the revelation of these milestone findings, scientists all over the world investigated the roles of Isl1 in cardiovascular development in more details. In contrast to the roles of Isl1 in mouse hearts which is essential for the development of both arterial and venous poles (Cai et al., 2003; Evans et al., 2010), in zebrafish, isl1 mutants showed defects only in the venous pole (De Pater et al., 2009; Witzel et al., 2017). This phenotype was further examined by clarification of the specific functions of islet family members in zebrafish cardiogenesis (Witzel et al., 2017). Different from the situation in mouse, where only one islet family member (Isl1) plays vital roles in embryonic heart development, in zebrafish, there are three islet family members (isl1, isl2a, and isl2b) involved in cardiogenesis (Witzel et al., 2017). In details, isl1 is required for the venous pole development; isl2b controls the development of the arterial pole; whereas isl2a plays a role in cardiac looping (Witzel et al., 2017). Moreover, in zebrafish, isl1 mutants showed bradycardia and arrhythmia phenotypes (De Pater et al., 2009; Tessadori et al., 2012), implying a role of isl1 in cardiac conduction. Later on, two studies provided crucial support for this conclusion by identifying that Isl1 is an important transcriptional regulator for pacemaker development and function in mice (Liang et al., 2015; Vedantham et al., 2015).

### The Cellular and Molecular Modes of Action of Isl1

After the role of Isl1 in cardiovascular development was clarified, interest was focused on the molecular mechanism of Isl1 action in CPCs. A number of factors downstream and upstream of Isl1 have been identified in cardiovascular development.

Mef2c was the first identified direct downstream target of Isl1 in the anterior portion of the SHF (known as the anterior heart field (AHF), or anterior SHF) (Dodou et al., 2004). Evidence showed that Isl1 bound directly to the two conserved consensus elements within a cardiac enhancer upstream of the first translated exon of mouse Mef2c (Dodou et al., 2004). Isl1 consensus binding sites were also identified in the first intron of Myocd (Kwon et al., 2009), a potent transcriptional co-activator of serum response factor regulation of smooth muscle and cardiac gene expression (Miano, 2015) and Fgf10 (Golzio et al., 2012; Watanabe et al., 2012), a secreted member of the fibroblast growth factor family, characterizing the pharyngeal mesoderm of the anterior SHF (Kelly et al., 2001; Watanabe et al., 2012). Direct occupation of Isl1 on both genes was demonstrated by ChIP and EMSA assays (Kwon et al., 2009; Golzio et al., 2012; Watanabe et al., 2012). In agreement with this, the levels of Myocd and Fgf10 were significantly reduced in Isl1 KO CPCs (Cai et al., 2003; Kwon et al., 2009). Moreover, Hand2 was found to be a direct downstream target of Isl1/Ldb1 complex in the SHF progenitors, with several Isl1 consensus binding sites in its proximal promoter and the OFTRV enhancer (Caputo et al., 2015).

There is no doubt that emerging molecular biology techniques will help in understanding the molecular mechanisms of Isl1 in cardiovascular development. With the help of high-throughput sequencing techniques, a genome-wide downstream targets network orchestrated by Isl1 was uncovered (Gao et al., 2019). This Isl1 regulatory network contains three main clusters of Isl1 primary downstream targets: 1) Transcription factors and epigenetic modifiers, such as some known Isl1 downstream targets, Mef2c, Myocd and Hand2, as mentioned above, as well as other key regulators of cardiac development, such as Gata4, Tbx20, Baf60c (Smarcd3); 2) Signaling molecules, such as Fgf10, which is also a known Isl1 target, and Bmp4, which was confirmed later in primates as reported recently (Yang et al., 2021); 3) Heart contraction and cardiomyocyte structural genes, such as Ttn, Ryr2, and Myl3 (Gao et al., 2019).

**FIGURE 1:**
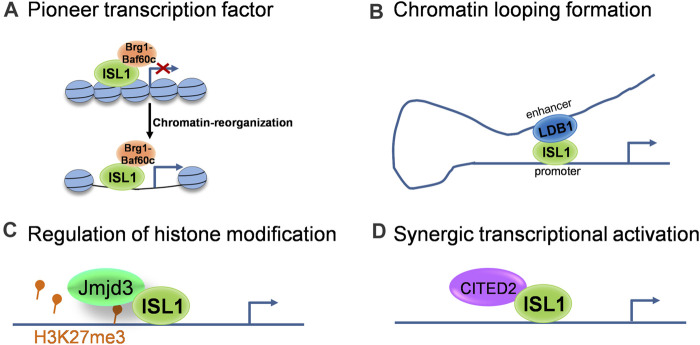
The molecular modes of action of ISL1 during cardiovascular development. **(A)** ISL1 acts as a pioneer transcriptional factor to shape the chromatin landscape. **(B)** ISL1 promotes chromatin looping formation via interaction with LDB1. **(C)** ISL1 is involved in the regulation of histone modification through interactions with histone modifiers. **(D)** ISL1 regulates target genes synergistically with transcriptional co-regulators.

As a transcription factor, the classical function of Isl1 is to bind specific DNA sequences in enhancer and/or promoter regions of its target genes and regulate their transcription. Recent studies revealed multifaceted roles of Isl1 on epigenetic and transcriptional regulation. Here we outline several modes of action of Isl1 reported in cardiovascular development (Figure 1): 1) Acting as a pioneer transcription factor to shape the chromatin landscape. Pioneer factors are a specialized subset of transcription factors, with the capacity to bind specific DNA sequences within closed chromatin and then trigger the remodeling of chromatin landscape changes to provide accessibility to non-pioneer transcription factors (Mayran and Drouin, 2018). Pioneer factors play critical roles in embryogenesis and cell fate specification (Zaret, 2020). Isl1 was recently identified to act as a pioneer factor in cardiogenesis, which works in concert with Brg1/Baf60c complex in CPCs to shape the chromatin landscape, thus regulating the expression of its downstream target genes (Gao et al., 2019). 2) Regulating target genes together with transcriptional and epigenetic co-regulators. As a pioneer factor, the pioneer activity of Isl1 and its subsequent role as a classical transcriptional regulator are implemented largely through its interactions with co-regulators (Zaret and Mango, 2016). For example, Isl1 forms a complex with Ldb1 in CPCs, promotes chromatin looping formation by bringing together distant enhancers and promoter elements, contributing to 3-dimensional chromatin reorganization at its target locus, thus driving CPC differentiation and SHF development (Caputo et al., 2015). Furthermore, Isl1 works synergistically with Jmjd3 in CPCs to promote the demethylation of H3K27me3 at the enhancers of key downstream target genes, instructing gene expression changes that drive cardiac differentiation (Wang et al., 2016). Transcriptional co-regulator CITED2 was also described in CPCs to cooperate with Isl1 to promote ESC differentiation toward cardiomyocytes (Pacheco-Leyva et al., 2016).

Overall, the sophisticated regulation of Isl1 during cardiovascular development much depends on its association with co-factors that work together in a context-dependent manner, which helps to explain how Isl1 regulates its target genes in different tissues to keep their specific expression pattern. So far, there are still very few Isl1 cofactors identified in cardiogenesis. More investigations need to be done to uncover the Isl1-interaction network in the cardiovascular context, which will help us to better understand the fine regulation of Isl1 on cardiovascular development.

### Regulation on Isl1

In contrast to its downstream regulation, little is known about the upstream regulation on Isl1 gene expression. Only some candidates were determined as Isl1 direct upstream regulators, including ß-catenin (Lin et al., 2007), TCF/LEF1 (Lu et al., 2014), Nkx2.5 (Dorn et al., 2015) and Forkhead transcription factors (Kang et al., 2009).

**FIGURE 2:**
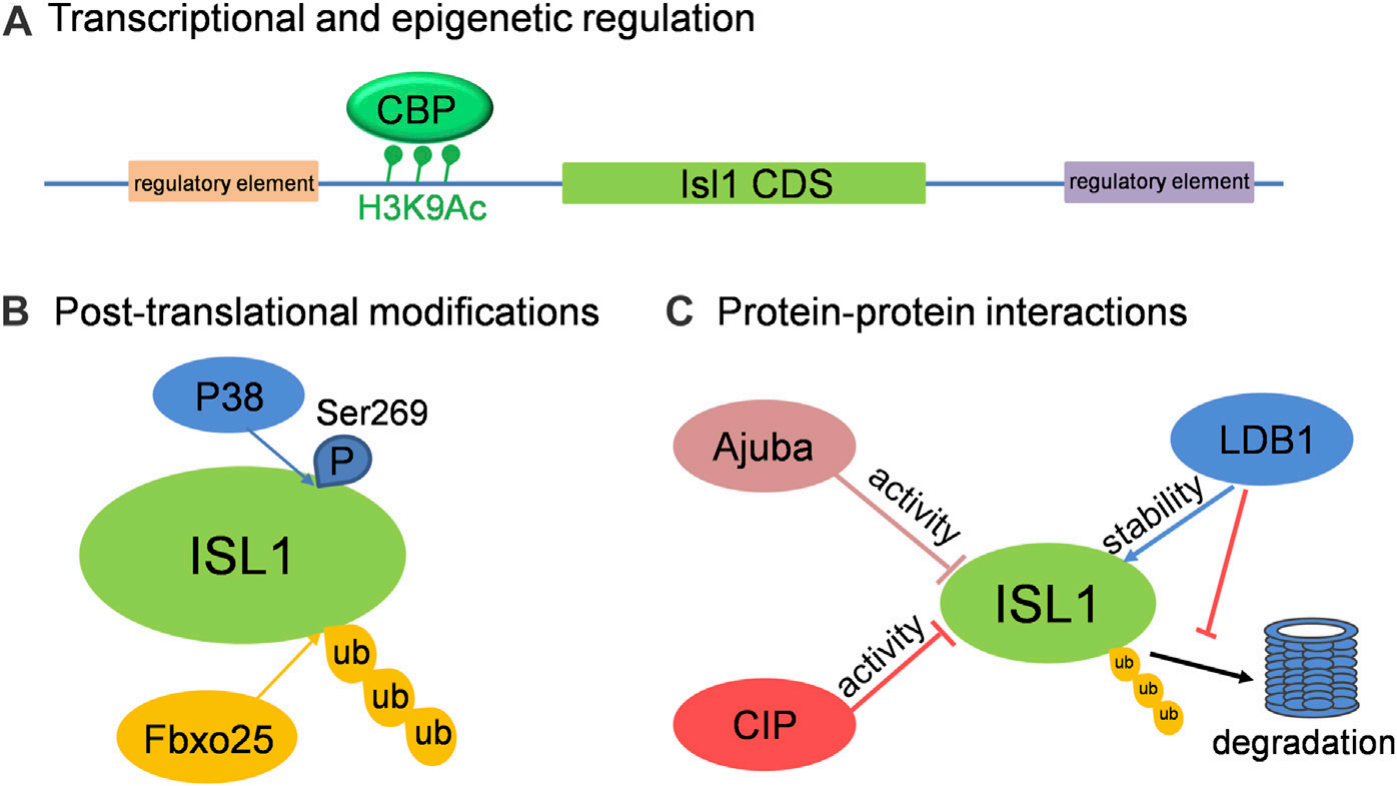
The cellular and molecular regulation on Isl1 in cardiovascular development. **(A)** Isl1 can be regulated at transcriptional level by binding of regulators on the regulatory elements located up- or down-stream of Isl1, or by histone modification on Isl1 promoter region. **(B)** ISL1 can be ubiquitinated by Fbxo25 and phosphorylated by P38. **(C)** The transcriptional activity and stability of ISL1 can be regulated by its interaction partners, such as CIP, Ajuba and LDB1.

In addition to those known upstream regulators of Isl1, there are still many factors or regulatory elements associated with the cellular and molecular regulation of Isl1 in different aspects, including the transcriptional activity and protein stability of Isl1. Three primary modes of regulation on Isl1 are highlighted here (Figure 2): 1) Transcriptional and epigenetic regulation: In a screening of regulatory elements in the Isl1 locus, an enhancer of Isl1 gene located downstream of Isl1 was identified. Studies with mice exhibited that this enhancer is involved in Isl1 regulation in the developing heart and dorsal aorta *in vivo* (Kappen and Salbaum, 2009). A 2.9-kb regulatory element that regulates pacemaker cell development and SAN function was also identified in the upstream of Isl1 with ATAC-seq in purified pacemaker cells and right atrial cardiomyocytes of neonatal mice (Galang et al., 2020). In addition, Wnt/ß-catenin signaling regulates Isl1 not only through binding of TCF/LEF1 on Isl1 promoter, but also promotes H3K9 acetylation conducted by CBP on TCF/LEF1 binding sites, resulting in upregulation of Isl1 expression in early stages of cardiomyocyte differentiation of P19CL6 cells (Lu et al., 2014). 2) Post-translational modifications: Fbxo25 was reported to act as an E3 ligase to target Isl1, as well as Nkx2.5 and Hand1, for ubiquitination and facilitate protein degradation of these cardiac transcription factors (Jang et al., 2011). More recently, Isl1 was shown to be phosphorylated by p38 MAPK at serine 269, which prevents Isl1 degradation and ensures its transcriptional activity during cardiogenesis downstream of BMP signaling (Jing et al., 2021). 3) Protein-protein interactions: Several proteins were identified to regulate the transcriptional activity or stability of Isl1 via interactions between them. CIP, co-expressing with Isl1 in CPCs of embryonic heart, negatively regulates the transcriptional activity of Isl1 via direct interaction (Huang et al., 2012). Similarly, Ajuba also binds Isl1 in CPCs and represses its transcriptional activity, thus restricting the SHF progenitor pool in zebrafish (Witzel et al., 2012). Ldb1, not only orchestrates cardiac-specific chromatin organization together with Isl1, but also binds Isl1 to protect it from degradation (Caputo et al., 2015). Taken together, the regulations on the expression, stability and activity of Isl1 are under tight control and in a context-specific manner to enable Isl1’s precise cellular and molecular functions in cardiovascular development.

### Islet1 and Congenital Heart Diseases

ISL1 variants/mutations have been found to be associated with congenital heart diseases (CHD). ISL1 rs1017 is one of the most frequently reported ISL1 variants; however, its correlation with CHD remains controversial. ISL1 rs1017 was reported to be related to the risk of CHD in a white population (Stevens et al., 2010) and a Chinese cohort (Luo et al., 2014), while the other studies showed opposite results (Xue et al., 2012; Cresci et al., 2013). Moreover, ISL1 haploinsufficiency was shown to be associated with d-transposition of the great arteries (d-TGA) (Osoegawa et al., 2014). Consistently, ISL1 heterozygous mutations (p.E137X and p.Tyr75*) were identified in an index of CHD patients (Ma et al., 2019; Wang et al., 2019). Altogether, these studies implied a genetic basis of ISL1 in the formation of CHD.

Given the critical roles of ISL1 in cardiovascular development, loss-of-function mutations of ISL1 would lead to severe cardiac malformations. So far, there are few ISL1 mutations reported in human CHD cases, which probably can be explained by early embryonic lethality of Isl1 inactivation and no gross phenotype of heterozygous Isl1 mutants as shown in mouse model (Pfaff et al., 1996; Cai et al., 2003). More recently, a study in non-human primate (NHP) uncovered a critical role of ISL1 in controlling mesoderm formation via signaling from the amnion, leading to early embryonic lethality in ISL1 mutant NHP embryos (Yang et al., 2021), providing a new insight for the roles of ISL1 in early embryogenesis.

## Conclusion

Cardiovascular disease remains one of the leading causes of mortality all over the world. Studies from multiple research groups have established promising strategies to trigger cardiac repair and heart regeneration, including direct reprograming non-myocytes to cardiomyocytes, activation of cardiomyocyte proliferation, as well as stem-cell based therapies (Cahill et al., 2017; Chien et al., 2019). ISL1^+^ CPCs derived from pluripotent stem cells are actively investigated for cardiac repair in recent years (Bartulos et al., 2016; Li et al., 2017; Wang et al., 2017; Foo et al., 2018; Ghazizadeh et al., 2018). Defining the molecular mechanisms underlying normal cardiovascular development is a prerequisite for understanding the etiology of cardiovascular diseases. In this review, we provide insights into Isl1 biology illustrated in current literature that sheds light on the prominent and multifaceted roles of Isl1 in cardiovascular development. Even though the gene regulatory network of Isl1 and its functions in cardiovascular development have been uncovered, the great details underlying its cellular and molecular modes of action still need further investigations. In addition, there are still challenges to integrate the roles of Isl1 to the comprehensive regulatory networks orchestrating cardiovascular development. With the development of new techniques and the implement of emerging powerful tools of genetic studies such as single-cell RNA sequencing and CRISPR-Cas9 genome editing technique, the detailed behavior of Isl1 and the molecular mechanisms controlling cardiovascular development will be further elaborated, and more insights will be provided for the development of novel strategies for cardiac repair.
